# Sustainable Engagement and Academic Achievement Under Impact of Academic Self-Efficacy Through Mediation of Learning Agility—Evidence From Music Education Students

**DOI:** 10.3389/fpsyg.2022.899706

**Published:** 2022-06-14

**Authors:** Zhang Jian

**Affiliations:** College of Fine Arts, Henan University of Economics and Law, Zhengzhou, China

**Keywords:** sustainable performance, learning agility, self-efficacy, academic achievement, motivation

## Abstract

The overarching goal of this study was to look into the effects of academic self-efficacy and academic motivation on student long-term engagement and academic achievement. This study also sought to investigate the role of learning agility as a mediator in the relationship between academic self-efficacy and academic motivation. This study examined the impact of student sustainable engagement on student academic achievement as part of our model. A questionnaire technique was used to collect data from 325 music education students studying at various music training institutions in China. The data were analyzed using the Smart-PLS software and a structural equation modeling (SEM) technique. Academic self-efficacy and academic motivation were found to have a positive and significant relationship with student long-term engagement. The academic motivation was also found to have a positive relationship with student long-term engagement. Furthermore, learning agility was found to mediate the relationship between academic self-efficacy and student sustainable engagement. Furthermore, learning agility mediated the relationship between academic motivation and long-term student engagement. Furthermore, student sustainable engagement has a significant and positive relationship with student academic achievement. This paper made a valuable theoretical contribution by investigating the impact of academic self-efficacy and academic motivation on student sustainable engagement, as well as the impact of student sustainable engagement on student academic achievement. Furthermore, this study added to the body of knowledge by investigating the relationship through the lens of cognitive learning theory. In terms of practical implications, this paper would undoubtedly assist educational institutions in maintaining a fair and just learning environment that encourages students to be engaged and perform well. Future research can include other constructs to gain a better understanding of the factors that influence students’ academic engagement and achievement.

## Introduction

Sustainable student engagement in music education has a number of cognitive, interpersonal, and psychological advantages, including emotional regulatory control, self-identity generation, and social interaction improvement (Weinberg and Joseph, 2016). Awareness of the benefits of music education engagement, multiple studies, and reports have found that student engagement in music activities in industrialized countries is small in comparison to certain other school disciplines and that students’ value of engagement in music declines over the high school years (McPherson and O’Neill, 2010). The reason for such a disengagement in music education might be the focus placed on teaching Western “classical” music notation in many school music programs which frequently excludes other musical forms and practices (Richmond et al., 2015).

Even if instructors provide a variety of musical forms and activities, students must find these practices personally valuable in order to retain their engagement in music education over time (López-Íñiguez and Bennett, 2020). Effective music engagement emphasizes on participating in a training program by students who have a thorough grasp and appreciation of the practice, out of which they receive a feeling of relevancy, goal, and satisfaction (Chen and O’Neill, 2020). Student engagement is a creative activity that occurs as a result of the ongoing symbiotic association between enthusiasm and discovery learning. Students’ active engagement in music activities is influenced by the value attributed to the activity (Fredricks et al., 2016).

Student engagement has also been linked to a reduction in criminality, substance abuse, and unhappiness (Wang and Fredricks, 2014; Fredricks et al., 2016). There is evidence that engagement is pliable and receptive to adjustments in educators’ and institutions’ approaches, which makes it desirable. Therefore, engagement has enormous potential as a significant focus for reforms and is a stated objective of several school development initiatives at the upper-secondary level. According to research, students are more engaged in classrooms in which they have got good relationships with their peers and teachers; where instructors promote students’ independence; where instructors have lofty aspirations and provide clear and consistent feedback; or where tasks are varied, daunting, fascinating, and impactful (Fredricks et al., 2016). Various nested settings, such as pro-social organizations, institutions, schools, pedagogical practices, and timescales, have been explored (shorter and longer engagement). As different studies have different definitions of engagement, many academics believe that motivation and engagement remain linked but are distinct notions (Groccia, 2018). The rise in popularity of participation in the study, administration, and practice may be attributed to a number of factors. So, to begin with, engagement is an important factor in students’ academic achievement. Student involvement has been connected to the reward system, achievement grades, and education completion rates in an increasing body of information (Wang and Fredricks, 2014).

This pertains to a student’s assessment of his or her capacity to engage in and accomplish academic assignments effectively. It can be argued that a sense of self-efficacy enables a person to trust in his abilities to withstand obstacles that prevent him from achieving his goals. The students’ academic self-efficacy allows them to achieve their objectives and ambitions (Hussain et al., 2021). Motivation is impacted by some contextual factors: external stimuli, temperament, objective, and instruments for achieving goals. Individuals develop sufficient motivation in order to attain their goals, desires, and inclinations (Mizumoto, 2013). Motivation for academic achievement is particularly important in the case of students. Individuals are motivated to finish an activity, achieve a goal, or get a certificate of competence in their respective careers when they are motivated in this way (Li et al., 2021). Resultantly, motivation explains the reasons for people’s actions and dictates why they act in a certain way. Motivation-driven actions are energizing, goal-oriented, and long-lasting. Motivation does have a multi-faceted structure in education, and it is linked to learning and academic motivation (Li et al., 2021). Academic motivation and sustainable student engagement, two of the most important examples of better educational behaviors, play a key role in students’ academic achievement. As a result, one of the main responsibilities of all successful educators is to increase students’ academic motivation and engagement (Peng, 2021).

Learning agility is becoming more universally acknowledged in managing people as a critical component of long-term instructional leadership (Murphy, 2021). Individual learning agility is the best predictor of future performance. Learning agility is described as an individual’s capacity and willingness to learn new skills under a variety of circumstances in the first place. Deep learning agility in many settings can provide new experiences (Murphy, 2021). Therefore, we suggested that learning agility could mediate the relationships between academic self-efficacy, academic motivation, and sustainable student engagement, leading to academic achievement. Academic achievement is not itself a determiner of students’ performance, while many studies in past have suggested that different kinds of performances refer to the academic achievement of students, such as class performance, test performance, and composite performance as an aggregated result. This cannot be individually evaluated without going into the exploration of contributors to academic achievement (Madigan and Curran, 2021). Several researchers evaluated different indicators and contributors to academic achievement, including grade point averages, class performance, perceptions about evaluation, etc. (Schneider and Preckel, 2017). A lot of other studies revealed the connection between student engagement and academic achievement and also demonstrated that academic achievement is linked to student engagement (Alhadabi and Karpinski, 2020).

It was also suggested that student engagement is linked to several contributing factors, among which motivation is the strongest contributor to students’ engagement. Several authors identified that although motivation and engagement are linked, they should be considered distinct concepts (Groccia, 2018). These studies were mostly conducted at college and higher secondary school levels. This whole gap in previous research allowed the authors to find and devise a conceptual model in which relationships between these variables could be studied in the field of music education at higher levels of educational institutions. Some questions were raised: How academic self-efficacy could lead to sustainable student engagement and influence academic achievement? How students’ motivation could contribute to sustainable students’ engagement leading to academic achievement? and What role learning agility could play in aiding these relationships? This research would help in providing answers to these questions.

## Theoretical Underpinning

Social Cognitive Theory is undoubtedly the most well-known of the several theories that attempt to describe the factors that control and govern behaviors (Bandura, 1977). According to Social Cognitive Theory, behavior is driven and regulated by a variety of social systems and related personality elements. Self-efficacy (SE) seems to be a fundamental component of these self-influence elements, and it pertains to a participant’s assessment of their ability to plan and execute the actions necessary to attain intended results (Bandura, 1997). The impact of self-efficacy on cessation of smoking, nutritional change in behavior, addiction recurrence, job conduct, sports ability and achievement, and academic success has been examined across a range of psycho-social fields (Honicke and Broadbent, 2016). Self-efficacy beliefs, according to Bandura (1997), are self-regulatory and important to human functioning because they give people the ability to influence their own cognitive processes and actions, and hence modify their surroundings.

Self-efficacy is defined as “People’s judgments of their skills to plan and courses of action required to achieve specific kinds of outcomes.” Self-efficacy is essential for students to continue to perform challenging tasks. SE is usually articulated in academic contexts under the perspective of academic self-efficacy (ASE), which refers to learner assessments of their ability to achieve educational goals (Putri and Prabawanto, 2019). This led us to utilize academic self-efficacy in the context of academic achievement through sustainable student engagement. Almeida (2021) categorized learning approaches into two categories, deep and surface learning approaches, while presenting a valid theory of learning in formal educational settings. Learners who are engaged in the deep learning strategy attempt to integrate new knowledge by connecting it to the individual perspective, background knowledge, and material. In contrast, in surface learning, learners place a greater emphasis on memorization and rote learning rather than appreciating the true significance of themes and theories (Coertjens et al., 2016). Test anxiety or the need to complete course standards in order to pass tests are common motivations for these kinds of learning, and learners aim to achieve these goals with the least amount of work possible (Mahmood et al., 2021). Expectancy Value Theory explains the relationship between self-efficacy and engagement. People’s behavioral and emotional engagement is impacted by their degree of self-efficacy (Mahmood et al., 2021). All these theories provide the basis for the inter-connected phenomenon of academic achievement. Academic self-efficacy and motivation leading to sustainable student engagement in music education which in turn leads to academic achievement are goals of this study supported through the above-mentioned theories.

### Academic Self-Efficacy and Sustainable Student Engagement

Academic self-efficacy (ASE) refers to a set of attitudes and judgments about a student’s efficacy and capacity to carry out specific educational tasks and responsibilities (Fife et al., 2011). Academic self-efficacy is defined by academicians as a student’s assessment of his or her ability to achieve academic objectives. Academic self-efficacy has been demonstrated to consistently link favorably with learning achievement and efficient methods of coping, according to a slew of investigations (Paciello et al., 2016). Students with stronger academic self-efficacy have already been observed to handle difficult academic tasks and their educational lives more efficiently (Allari et al., 2020). Children with poor academic self-efficacy are also more susceptible and less eager to execute academic tasks, and being less inspired, avoid school and experience stress and academic disappointment (Bassi et al., 2007).

Although there are few studies on the relationship between academic self-efficacy and study engagement, empirical investigations on students’ self-efficacy and other engagement themes exist, and a large number of studies show significant and favorable results (Olivier et al., 2019). ASE and learning engagement are being investigated, and it was found that ASE and learning engagement were linked, according to a previous study (Zhen et al., 2017). Students with ASE reported better academic ambitions by sitting down on assignments and engaging in learning activities, and a similar observation was reported in another research published by Bassi et al. (2007) among teenagers. In a study among university students in Japan (2013), Mizumoto, discovered that students with high academic self-efficacy were more enthusiastic about adopting vocabulary acquisition tactics than those with low to moderate ASE. All the suggested literature allowed us to devise the following hypothesis in our study context.

***H*_1_**. *Academic self-efficacy shows a connection with sustainable student engagement.*

### Academic Motivation and Sustainable Student Engagement

Motivation can be thought of as the driving factor which inspires learners to focus on academics and gain better achievements or grades in an academic setting. The academic motivation was classified into two parts by researchers: internal motivation and external motivation. Internal motivation is a relatively high element wherein identity, ambition, and pleasure guide conduct. Motivation can be described as behavior that is motivated by other objectives or incentives other than the activity itself. Within that case, the achievement of a specific activity is linked to external incentives or penalties, rather than the task itself or the individual. Whenever people do not really anticipate anything else in return for their actions, this is referred to be a motivating situation (Urdan and Bruchmann, 2018). Engagement, along with motivation, is seen as critical for improved learning outcomes for all the students in the literature. Student engagement in learning is considered a pre-requisite and a vital part of motivation (Schlechty, 2000). Engaging students in education is not just a means to an end. This is a goal in and of itself, even though it is a means to an end of students getting good academic results. It is significant since genuine engagement can contribute to better academic performance during a student’s academic career (Ryan and Deci, 2009). If instructors would like to know how to solve the challenges that young kids face, as well as make education more interesting, they must first listen to what students have to say about their classrooms and professors (Saeed and Zyngier, 2012). There are a few studies in the past that looked into the relationship between academic motivation and student engagement in different contexts (Peng, 2021). Therefore, the authors suggested the following hypothesis.

***H*_2_**. *Academic motivation shows a connection with sustainable student engagement.*

### Learning Agility

Learning agility is an essential component that blends efficacy and motivation into engagement in music education as a mediator between the ASE, academic motivation, and long-term student engagement. Humans live in a time when knowledge is shifting from predetermined skills and background information to optimal learning environment agility in unfamiliar circumstances using a variety of experiences plus technology instruments. Lombardo and Eichinger (2000) invented the phrase “learning agility.” This has been characterized as the motivation and capacity to learn through knowledge and then apply acquired information in new settings for good achievement as an educational technique based on past experience. This is defined as the capacity to learn new things on the move and the desire to put what you have learned into practice. It consists of three key elements: capacity, ambition, and adaptation (De Meuse et al., 2010).

Highly adaptable students can take the proper learning from their past experiences and use them in unfamiliar circumstances. Agile learners, according to De Meuse et al. (2010), can assess difficulties rapidly and precisely, synthesize knowledge, and grasp diversity. Learners are open to opportunities and situations, and their problem-solving methods are adaptable. Seemingly, agile learners are ready to learn, test hypotheses, and recognize learning in addition to increasing their abilities to deal with problems. Such learning agility will also be required to deal with problems in education and in future work environments (Murphy, 2021). Learning agility, as per De Meuse et al. (2010), is a rather stable notion that is independent of race, gender, or nationality.

This is due to the fact that learning agility is a better predictor of good success than cognitive or behavioral traits (Connolly, 2001). As a result, prior experiences with using and efficacy beliefs in students’ learning and lives have a role in modulating the impacts of ASE, academic motivation, and long-term student involvement as measured by learning agility. When students are faced with unclear situations, learning agility allows them to adapt and establish new values (Connolly, 2001). Previously, a few scholars evaluated different relationships in connection to learning agility as mediators, such as learning culture and work engagement (Saputra et al., 2018), and employee engagement through the learning culture of organizations (Tripathi and Sankaran, 2021). It indicated that learning agility being a mediator between ASE, academic motivation, and sustainable student engagement along with being an influencer of sustainable student engagement could be utilized, and we formulated the following hypothesis.

***H*_3_**. *Learning agility could be a mediator between academic self-efficacy and sustainable student engagement.*

***H*_4_**. *Learning agility could be a mediator between academic motivation and sustainable student engagement.*

***H*_5_. Learning agility could lead to sustainable student engagement.**

### Association of Student Engagement With Academic Achievement

Academic achievement has previously been considered a key result of student engagement. While there has been a large number of investigations on the topic, the findings have been divided. This connection may be seen from two different angles. For instance, Alhadabi and Karpinski (2020) discovered a considerable and reasonably strong link between the academic achievements of students and their engagement, while King (2015) discovered that academic achievement was favorably connected with emotional and behavioral engagement. A few researchers discovered a link between academic achievement and cognitive engagement (Pietarinen et al., 2014). Student engagement is thought to improve school achievement, which in turn encourages learners to participate in such activities, producing a “vicious circle of education” (Xu and Qi, 2019).

According to several experts, the positive relationship between behavioral engagement and achievement is stronger than between cognitive and emotional involvement. The findings of these studies suggest that various measures of student engagement show varying associations to academic achievement when considered altogether (Furrer and Skinner, 2003). Many investigators, on the other hand, did not achieve the same results, and in several investigations, there was no substantial link between academic achievement and student engagement. Shernoff and Schmidt (2007), for instance, discovered that among African-Americans, student participation did not predict grade point average. In addition, Konold et al. (2018) discovered no link between student academic achievement and engagement. Shernoff and Schmidt (2007) also said there was no link between students’ proactive emotional engagement and overall achievement levels in English and Maths. According to Appleton et al. (2006), the relationship between intellectual engagement and academic achievement is minimal. This literature suggested formulating the hypothesis for evaluating the relationship between sustainable student engagement and their academic achievement in the context of music education. Therefore, the authors proposed the following hypothesis.

***H*_6_**. *There is a strong association between sustainable student engagement and their academic achievement.*

**FIGURE 1:**
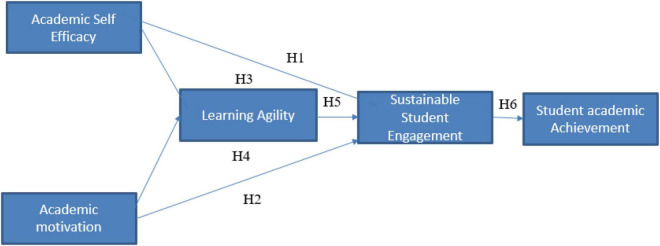
Conceptual framework.

The following conceptual model (Figure 1) has been formed based on the above findings and hypotheses.

## Methodology

The current study took a quantitative method with a deductive approach, in which hypotheses were formed and analyzed to see how specific variables affected other variables. This methodology is used by researchers to ensure that there is no bias. Self-administered surveys were utilized to obtain data for the analysis of this quantitative study. The music students from different institutes in China worked as the study’s target group. The convenience sample strategy was used to collect data from respondents *via* physically distributed questionnaires in this study. The data collection and process completion took 2–3 months. The overall number of questionnaires circulated was approximately 500, and we received 350 responses, resulting in a 70% response rate; out of those 530 responses, 325 were viable and used for data analysis. Because the data gathering procedure was delayed at the beginning of the study, reminders were sent to the selected responders to speed up the process. The students of music education in China served as our study unit of analysis.

### Statistical Tool

The Smart-PLS 3 was used to analyze the data in this study. The method used in our study was structural equation modeling. Partial least square is widely used in management and social sciences, because it is a variance-based structural equation modeling technique (Nitzl et al., 2016). Furthermore, PLS-SEM is a causal modeling technique whose goal is to increase the explained variance of latent dependent constructs. PLS-SEM is viewed by researchers as a “silver bullet” for dealing with empirical findings with small sample size. Smart-PLS is simple to use and has a plethora of advanced features (Garson, 2016). Furthermore, the Smart-PLS technique is best suited for research studies with complex equations (Wong, 2013). To precisely calculate the values of beta, reliability, and standard error, this study adheres to the recommendations of Wong (2013), and ensures that all of those indicators are part of their respective latent variables with outer loadings of 0.7 in the reflective outer model evaluation.

### Measurement

To record respondents’ responses, a five-point Likert scale ranging from strongly agree to strongly disagree was used in this study. The Cronbach’s alpha value should be greater than 0.7 when checking the reliability of each variable (Hair et al., 2019). We have included the measurement and the Cronbach’s alpha values in the following text:

#### Academic Self-Efficacy

This study utilized the scale of Wang et al. (2022), which is a single-dimension three-item scale. The value of Cronbach’s alpha is 0.826, which is acceptable when compared to the benchmark value.

#### Academic Motivation

The academic motivation was measured by using the scale of Vallerand and Blssonnette (1992) which consists of 14 items. The value of Cronbach’s alpha is 0.919, which is well above the required value.

#### Learning Agility

In this study, learning agility was measured by using a five-item scale by Gravett and Caldwell (2016). The Cronbach’s alpha value of learning agility is 0.905, which is acceptable as compared to the benchmark value.

#### Student Sustainable Engagement

This study measured student sustainable engagement by utilizing the scale of Briggs and Towler (2005), which consists of five items. The Cronbach’s alpha is 0.919, which is well above the benchmark value.

#### Student Academic Achievement

This study utilized student academic achievement by using the 19-item scale of Topor et al. (2010), while our study adopted 15 items for the purpose of our study. The Cronbach’s alpha is 0.92 which is acceptable.

### Demographic Analysis

The demographic profile of the respondents who took part in the survey is presented in Table 1. There were 325 people in total that took part in the study, with 189 men and 136 women. Bachelor’s degree holders made up 65% of the overall number of participants, while undergraduates constituted 35%. To acquire data in a cost-effective and time-saving manner, respondents were chosen using the convenience sampling technique.

**TABLE 1:** Demographic information.

Variable	Groups	No of respondents	Total
**Gender**	Male	189	325
	Female	136	
**Education**	Undergraduate	211	
	Graduate	114	325

### Common Method Bias

Table 2 displays the overall variation explained for each of the variables studied using single-factor analysis. It discusses the usual technique bias or the questionnaire’s bias. For one item, the percent of variance must be less than 50% (Yong and Pearce, 2013). Because the total variation explained in this study is less than 50%, there is no bias in the data.

**TABLE 2:** Common method biasness.

**Component**				**Extraction sums of squared loadings**		
	
	**Total**	**% of variance**	**Cumulative %**	**Total**	**% of variance**	**Cumulative %**
1	28.463	47.438	47.438	28.463	47.438	47.438
2	4.474	7.456	54.894			
3	2.309	3.849	58.743			
4	1.693	2.821	61.564			
5	1.45	2.416	63.98			
6	1.236	2.059	66.04			
7	1.175	1.958	67.997			
8	1.036	1.727	69.725			
9	0.999	1.666	71.391			
10	0.939	1.564	72.955			
11	0.862	1.436	74.391			
12	0.757	1.262	75.653			
13	0.719	1.198	76.851			
14	0.683	1.138	77.99			
15	0.636	1.06	79.05			
16	0.606	1.01	80.06			
17	0.593	0.989	81.049			
18	0.581	0.968	82.017			
19	0.558	0.93	82.947			
20	0.53	0.884	83.83			
21	0.521	0.869	84.699			
22	0.511	0.851	85.55			
23	0.47	0.784	86.333			
24	0.446	0.743	87.077			
25	0.432	0.72	87.797			
26	0.427	0.711	88.508			
27	0.402	0.671	89.179			
28	0.386	0.644	89.823			
29	0.359	0.598	90.421			
30	0.349	0.581	91.002			
31	0.342	0.57	91.572			
32	0.318	0.53	92.102			
33	0.314	0.523	92.625			
34	0.303	0.505	93.13			
35	0.293	0.488	93.619			
36	0.281	0.468	94.087			
37	0.273	0.455	94.541			
38	0.257	0.429	94.97			
39	0.246	0.411	95.38			
40	0.237	0.395	95.775			
41	0.22	0.367	96.142			
42	0.214	0.356	96.499			
43	0.196	0.327	96.826			
44	0.187	0.312	97.138			
45	0.183	0.305	97.443			
46	0.172	0.287	97.73			
47	0.16	0.267	97.997			
48	0.156	0.259	98.257			
49	0.14	0.234	98.49			
50	0.135	0.225	98.715			
51	0.125	0.209	98.924			
52	0.124	0.206	99.13			
53	0.116	0.193	99.324			
54	0.102	0.17	99.493			
55	0.097	0.162	99.655			
56	0.088	0.147	99.802			
57	0.087	0.144	99.946			
58	0.017	0.029	99.975			
59	0.01	0.017	99.993			
60	0.004	0.007	100			

*Extraction method: principal component analysis.*

## Data Analysis and Results

### Measurement Model

**FIGURE 2:**
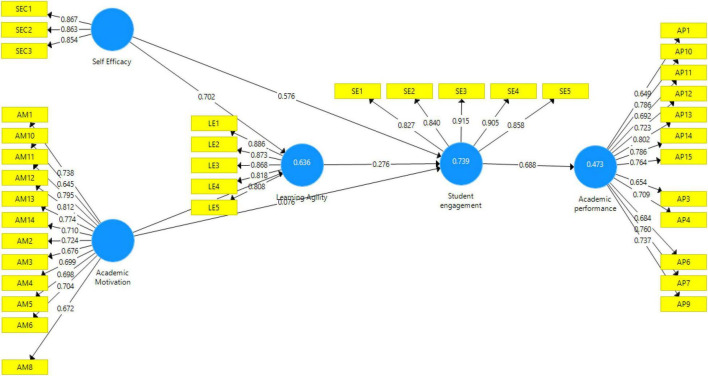
Measurement model.

The algorithm for the output measurement model is depicted in Figure 2. This diagram shows the effect of independent variables on the dependent variables of the study.

The table below shows the factor loadings for each study construct, namely, academic self-efficacy, academic motivation, learning agility, student sustainable engagement, and student academic achievement. Along with the VIF values, the table displays the composite reliability and average variance extracted (AVE). The factor loading explains an item’s contribution to the variable, and its value must be greater than 0.60 (Jordan and Spiess, 2019). The factor loadings for all the items in this study are greater than 0.60, indicating that the factor loadings are fair. The variation inflation factor (VIF) validates the model’s collinearity problems. The outer VIF result for the current study is also less than 5 (ranging from 1.804 to 4.211), indicating that there is no collinearity in the model. Furthermore, the current inner VIF result is less than 5 (between 1.508 and 2.475). Table 3 shows that the AVE values are greater than 0.60, indicating the presence of convergent validity (Ahmed and Azmi bin Mohamad, 2016). The composite reliability was greater than 0.70, putting it within the range of highly satisfactory values (Peterson and Kim, 2013).

**TABLE 3:** Reliability and validity.

Constructs	Items	Loading	Alpha	rho_A	CR	AVE	VIF
Academic motivation			0.919	0.926	0.929	0.522	
	
	AM1	0.738					3.103
	AM2	0.724					3.329
	AM3	0.676					2.528
	AM4	0.699					2.337
	AM5	0.698					2.189
	AM6	0.704					2.026
	AM8	0.672					1.74
	AM10	0.645					1.693
	AM11	0.795					2.705
	AM12	0.812					2.985
	AM13	0.774					2.182
	AM14	0.71					1.81

Academic performance			0.92	0.925	0.932	0.534	
	
	AP1	0.649					1.607
	AP3	0.654					1.996
	AP4	0.709					2.281
	AP6	0.684					1.892
	AP7	0.76					2.617
	AP9	0.737					2.145
	AP10	0.786					2.423
	AP11	0.692					1.835
	AP12	0.723					2.076
	AP13	0.802					2.464
	AP14	0.786					2.757
	AP15	0.764					2.475

Learning agility			0.905	0.909	0.929	0.724	
	
	LA1	0.886					3.37
	LA2	0.873					2.959
	LA3	0.868					2.75
	LA4	0.818					2.301
	LA5	0.808					2.179

Student engagement			0.919	0.921	0.939	0.756	
	
	SE1	0.827					2.139
	SE2	0.84					2.555
	SE3	0.915					4.018
	SE4	0.905					3.769
	SE5	0.858					2.745

Self-efficacy			0.826	0.827	0.896	0.742	
	
	SEC1	0.867					1.905
	SEC2	0.863					1.965
	SEC3	0.854					1.781

*AVE, average variance extracted; CR, composite reliability; AP, academic performance; AM, academic motivation; SEC, self-efficacy; SE, student engagement; LA, learning agility.*

The HTMT ratio and the Fornell and Larker criteria were used to assess discriminant validity (see Tables 4, 5). These tests determine whether or not there is a difference between the variables. According to Jordan and Spiess (2019), the HTMT rati should be less than 0.90 to ensure a variable’s discriminant validity. The HTMT ratio for the current study was less than 0.90, indicating that discriminant validity exists. The Fornell and Larker criteria are met if the value at the top of the column is greater than the value below that column (Henseler et al., 2015).

**TABLE 4:** Fornell and Larcker criteria.

	AM	AP	LA	SEC	SE
AM	0.722				
AP	0.692	0.731			
LA	0.558	0.725	0.851		
SEC	0.584	0.699	0.788	0.861	
SE	0.566	0.688	0.773	0.838	0.870

*AP, academic performance; AM, academic motivation; SEC, self-efficacy; SE, student engagement; LA, learning agility.*

**TABLE 5:** Heterotrait–Monotrait ratio.

	AM	AP	LA	SEC	SE
AM					
AP	0.711				
LA	0.571	0.788			
SEC	0.629	0.796	0.815		
SE	0.572	0.740	0.844	0.821	

*AP, academic performance; AM, academic motivation; SEC, self-efficacy; SE, student engagement; LA, learning agility.*

An *R*-square value greater than or close to 0.50 indicates that the model is substantial and good (Archer et al., 2021). The *R*-square values for the current study’s variables are close to or greater than 0.50, indicating that the model is good. The cross-validated redundancy should be greater than zero as measured by *q*-square (Henseler et al., 2015). The *Q*-square values for the current study’s variables are greater than zero, indicating that the model is significant as shown in Table 6.

**TABLE 6:** *R*-square values and *Q*-square values for the variables.

Constructs	*R* ^2^	*Q* ^2^
Academic performance	0.473	0.215
Learning agility	0.636	0.317
Student engagement	0.738	0.418

### Structural Model

**FIGURE 3:**
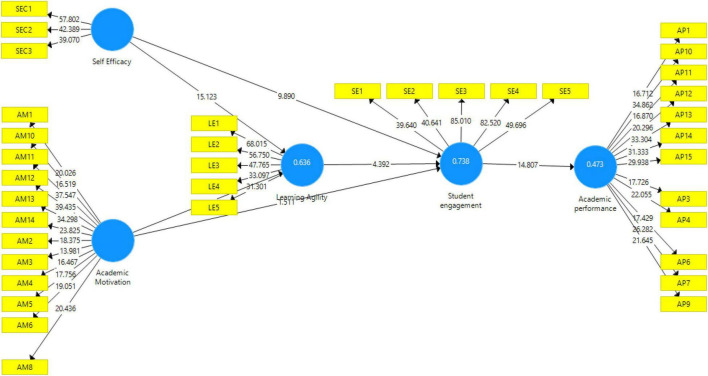
Structural model.

After successfully analyzing the measurement model, which includes the establishment of constructs and indicator reliability and validity, in the next step, a structural model was assessed to measure the coefficient of determination (*R*^2^), significance of path coefficient, and relevance of path coefficient (see Figure 3).

*R*-square explains the chance in endogenous variables happened due to exogenous variable. Recommended value of (*R*^2^) should be greater than 0.10. *R*-square value criteria proposed by Hair et al. (2013) includes 0.75, 0.50, or 0.25 for endogenous latent variables and can, as a rough rule of thumb, be described as substantial, moderate, or weak, respectively. *R*-square value of learning agility is 0.636, which shows that 63% of changes in the learning agility are accounted for because of academic self-efficacy and motivation. Similarly, *R*-square value of student engagement is 0.738 and academic achievement is 0.473, which shows that 73% variance in student engagement and 47% variance in academic achievement are accounted for because of exogenous variable in the model. *Q*-square is predictive relevance and measures whether a model has predictive relevance or not (>0 is good). *Q*^2^ values of all the endogenous constructs are greater than 0, which shows the predictive relevance of the model.

In the next step, the significance of the path coefficient and relevance of the path coefficient were measured to investigate the hypotheses of this study. The bootstrapping procedure was applied by using 5,000 bootstrap samples for the assessment of the path coefficient. Table 7 shows the direct effects of the variable under study, while Table 8 shows the indirect effects of the variable under study. These tables show whether the hypotheses were accepted or rejected based on the *p*-values that were less than 0.05 (Andrade, 2019).

**TABLE 7:** Summary of structural analysis (direct effect).

Hypotheses	Relationship	Beta	SD	*T* value	*P* values	Confidence interval		Decision
						LL	UL	
H1	Self-efficacy → Student engagement	0.575	0.058	9.890	0.000	0.467	0.695	Supported
H2	Academic motivation → Student engagement	0.075	0.050	1.511	0.131	-0.028	0.169	Unsupported
H5	Learning agility → Student engagement	0.277	0.063	4.392	0.000	0.711	0.828	Supported
H6	Student engagement → Academic performance	0.688	0.046	14.807	0.000	0.578	0.764	Supported

**TABLE 8:** Summary of mediation analysis.

Hypotheses	Constructs	Total effect		Indirect Effect		Confidence interval	
						**LL**	**UL**
H3	Self-efficacy → Learning agility → Student engagement	0.702	0.000	0.194	0.000	0.106	0.285
H4	Academic motivation → Learning agility → Student engagement	0.116	0.025	0.041	0.034	0.072	0.204

Hypothesis 1 (H1) proposed that there is a relationship between self-efficacy and student engagement. The results show that if self-efficacy increases by one unit of standard deviation, then the student engagement increases by 0.575 standard deviation units (*B* = 0.575, *T*-value = 9.890, *p* = 0.000). H2 proposes that there is a relationship between academic motivation and student engagement. The results indicate that academic motivation does not show a significant impact on student engagement at *P* > 0.005. Hence, H2 is rejected. H5 proposes that learning agility has a positive and significant impact on student engagement. The results show that learning agility has a positive and significant impact on student engagement (*B* = 0.277, *P* = 0.000) Hence, H5 is accepted. Moreover, H6 proposes that a relationship exists between student engagement and student academic achievement. The results reveal that student engagement has a positive and significant impact on student academic achievement. As student engagement increases by one standard deviation unit, the academic performance also increases by 0.688 standard deviation units. Hence, H5 of this study is accepted.

In this study, mediation analysis was performed by using the method proposed by Preacher et al. (2016). H3 proposes that learning agility mediates the relationship between self-efficacy and student engagement. The results show that learning agility partially mediates the relationship between self-efficacy and student engagement, as both direct and indirect effects are significant. H4 evaluates whether learning agility mediates the relationship between academic motivation and student engagement. The result shows that learning agility fully mediates the relationship between academic motivation and student engagement, as the direct effect between academic motivation and student engagement was insignificant, as shown in the Table 8.

## Discussion

The focus of this study was to evaluate the missing link between academic self-efficacy (ASE), academic motivation, and sustainable student engagement. The study also focused on evaluating the direct impact of sustainable student engagement on academic achievement, which was the ultimate goal of the research. This study also evaluated the mediator of learning agility between ASE, academic motivation, and sustainable student engagement.

According to the definition, ASE refers to different attitudes and behaviors about the self-efficacy of students for performing specific tasks related to education (Fife et al., 2011). It is defined by Elias and MacDonald (2007) as a method to assess the ability of students to perform and attain their goals and academic objectives. Previously, it has been considered for evaluating the academic achievements of the students and has shown a consistent link with coping mechanisms (Paciello et al., 2016). It was also observed that students having stronger academic self-efficacy were able to handle challenging academic tasks (Allari et al., 2020), whereas students with weak academic self-efficacy were less oriented to execute academic tasks (Bassi et al., 2007). Similarly, our results indicated that students who were more goal-oriented have greater academic self-efficacy, which contributed significantly toward sustainable student engagement in music education. These results are also in agreement with some of the previous investigations in similar contexts. It was evident from the literature review that there are a few studies on the relationship between academic self-efficacy and study engagement. Previously, the theme of the relationship between self-efficacy and student engagement does exist, indicating significant association (Olivier et al., 2019). Studies on ASE and learning engagement indicated that these two variables were linked (Zhen et al., 2017).

The second hypothesis of the current study examined the association between academic motivation and sustainable student engagement. Although some of the previous investigations indicated that students’ motivation is an indicator of student engagement, results of this study revealed contrasting results that academic motivation is not associated with sustainable student engagement. The possible reason for such outcomes lies in the fact that previously student engagement was assessed in a normal educational context, while this research evaluated the academic motivation in the context of music education and students could not be referred to as sustainably engaged in this context. It indicates that music could not establish its worth in educational institutes, even though sustainable student engagement has a lot to offer in music education, such as cognitive, interpersonal, and psychological advantages, including emotional regulatory control, self-identity generation, and social interaction improvement (Weinberg and Joseph, 2016). The other direct relationship between student engagement and academic achievement was also tested in this study, which proved that there was a significant association between sustainable student engagement and academic achievement. Academic achievement has previously been supposed as a promising indication of student engagement. Considering this fact, it was observed that there has been a large number of investigations on the topic. This kind of relationship was also studied by Alhadabi and Karpinski (2020) who identified a strong and considerable connection between the academic achievements of students and their engagement. However, some contrasting results were also produced where no connection was found between academic achievement and students’ engagement (Konold et al., 2018).

The indirect and mediating results of the current study revealed that there was a mediation of learning agility between ASE, academic motivation, and sustainable student engagement. The results proved their significance in a way that learning agility has been a helping tool in the education sector along with working scenarios of different organizations. This could be understood by the phenomenon of learning agility, according to which highly adaptable students can take the proper learning from their past experiences and use them in unfamiliar circumstances.

It is supported by the fact that learners who are agile in learning have the ability to assess the challenges quickly and from previous knowledge could adapt to the changes. This is also supported by the fact that the problem-solving nature of agile learners keeps them engaged, which ultimately leads to academic achievement (De Meuse et al., 2010). Therefore, similar results were obtained in evaluating the relationship of learning agility with students’ sustainable engagement. Some of the previous scholars also evaluated learning agility as a mediator in different contexts (Saputra et al., 2018; Tripathi and Sankaran, 2021). These scholars found the significant mediating role of learning agility in their studies.

### Practical Implications

This study provides several important recommendations for public policymakers and academic institutes which help to improve student engagement, which ultimately impacts student academic achievement. Teachers should provide positive moral inspiration to support students’ academic self-efficacy. Teachers should exhibit a favorable attitude in order to motivate students in the institutions. The curriculum should be designed by looking at the consideration of student motivation and self-efficacy. Special seminars and sessions should be conducted to boost student motivation and self-efficacy. The institution’s administration should pay attention to making academic institutions more collaborative and innovative to motivate students both intrinsically and extrinsically, which would further help to foster student engagement. The academic level of self-efficacy and academic motivation both contributed positively to the prediction of academic performance, according to the findings. As a result, students are expected to acquire experiences in school that will help them increase their academic drive and self-efficacy. Digital natives must be encouraged to identify suitable methods to use digital technology for academic success, rather than focusing solely on learning how to use digital technologies. As a result, digitally enhanced education for college students should presume that students can use technology to discover, analyze, produce, and share information, which should be employed in the context of academic problem-solving. Students’ future careers and quality of life will be influenced by their hands-on experience integrating digital technology with academic work.

### Theoretical Contribution

The findings imply that in order to achieve meaningful use of digital technology in academic life, students must make an effort to study and apply gained knowledge as agile learners in a digitally enriched environment. The findings support earlier research reports about the importance of motivation and self-efficacy in the student’s academic carrier. This study contributes to the body of research by exploring the two key predictors which lead to increased student engagement and academic achievement. This study also explores the mediating role of learning agility between motivation, self-efficacy, and sustainable student engagement. The findings on knowledge and skills and student engagement, in particular, suggest that digital competence has a favorable influence on student involvement, which is further connected to crucial outcomes, such as grades, perseverance, and college completion. Literature shows that limited studies explore the concept of learning agility. This study makes a unique contribution by exploring two important predictors which help to foster learning agility. Along with this, this study provides valuable insights for researchers and scholars to explore more antecedents that play a critical role in fostering student engagement, which impacts their academic performance and has a long-lasting impact on their careers.

### Limitations and Future Recommendations

This study found several shortcomings that must be addressed. One limitation of this study is that it included 325 students from varied backgrounds, including various professions, genders, and grade levels. The background of these students may indicate variations in student academic achievement. Second, our samples come from a particular area, so there is some issue of biasness here. To improve study generalizability, it would be preferable to increase the sample size; for example, future research may include students from several institutions in different cities to obtain a better model fit toward data and *R*-squared values for outcome variables. Third, data of this study were collected from only one single source, which may raise the risk of common method bias. So, future studies should collect data from multiple sources in order to avoid the risk of common method biasness. It is plausible that a number of limitations influenced the research findings, such as difficulties in assessing the student’s level of motivation, including a small proportion of students from few institutions without taking into consideration students from different countries, and including a small sample presented by students from a limited geographical area without giving due consideration to students from other countries. As a result, future research should collect data from other countries and do comparison analyses. They were equally significant in interpreting the results and planning future study possibilities. In this regard, it should be mentioned that while this study took into consideration previously acquired competencies as well as the observed outcomes of the e-learning course, modeling of the overall result was mostly dependent on the data provided by research participants themselves. This could have had an external validity. Even though the research did not take into account students’ specialization and attempted to provide as many similar contexts for respondents as feasible, future work of this type will give relevant comparisons.

## Conclusion

The prime objective of this study was to assess the key predictors that may help to facilitate student sustainable engagement and academic achievement. This study also investigated whether learning agility mediates the relationship between academic self-efficacy and student motivation on student engagement. Data for this study were collected from undergraduates and graduates studying in various colleges and universities in China. This study adopts the convenience sampling technique for the collection of data. A partial least square method was adopted for data analysis. Results reveal that intrinsic and extrinsic motivation is a pre-requisite for any student to set a career target and make their efforts to achieve it. If students have a strong belief in their skills and capabilities, then they will be more involved in academic activities and skill enhancement, which not only leads to an increase in their engagement with learning and education but also has a long-lasting impact on their future careers.

## Data Availability Statement

The original contributions presented in this study are included in the article/supplementary material, further inquiries can be directed to the corresponding author.

## Ethics Statement

The studies involving human participants were reviewed and approved by Henan University of Economics and Law, China. The patients/participants provided their written informed consent to participate in this study. This study was conducted in accordance with the guidelines of the Declaration of Helsinki.

## Author Contributions

ZJ conceived, designed, and wrote the manuscript, read, and agreed to the published version of the manuscript.

## Conflict of Interest

The author declares that the research was conducted in the absence of any commercial or financial relationships that could be construed as a potential conflict of interest.

## Publisher’s Note

All claims expressed in this article are solely those of the authors and do not necessarily represent those of their affiliated organizations, or those of the publisher, the editors and the reviewers. Any product that may be evaluated in this article, or claim that may be made by its manufacturer, is not guaranteed or endorsed by the publisher.
